# Anemia and its association with *Helicobacter pylori* infection among adult dyspeptic patients attending Wachemo University Nigist Eleni Mohammad Memorial Referral Hospital, Southwest Ethiopia: A cross-sectional study

**DOI:** 10.1371/journal.pone.0245168

**Published:** 2021-01-14

**Authors:** Kassahun Haile, Tilahun Yemane, Girum Tesfaye, Deneke Wolde, Abebe Timerga, Admasu Haile

**Affiliations:** 1 Departement of Medical Laboratory Science, Wolkite University, Wolkite, Ethiopia; 2 School of Medical Laboratory Sciences, Jimma University, Jimma, Ethiopia; 3 Departement of Medical Laboratory Science, Wachemo University, Hosanna, Ethiopia; 4 Department of Biomedical Science, Wolkite University, Wolkite, Ethiopia; University of Malaya Faculty of Medicine, MALAYSIA

## Abstract

**Background:**

Anemia is a worldwide public health problem and also associated with *Helicobacter pylori (H*. *pylori)* infection. Determining the association of anemia with *H*. *pylori* infection is important to develop evidence-based decision and intervention strategies, which is not well known in Ethiopia. Thus, this study aimed to determine the association between anemia and *H*. *pylori* infection among adult dyspeptic patients attending Wachemo University Nigist Eleni Mohammad Memorial Referral Hospital in Southwest Ethiopia.

**Methods:**

A cross-sectional study was conducted from January to April 2019 involving 362 consecutive adult dyspeptic patients who came to the hospital during the study period. Socio-demographic, clinical and other related data were collected by structured questionnaires. Four milliliters of the venous blood sample was collected for hematological parameters analysis and blood film preparation. A stool sample was collected to detect *H*. *pylori* antigen and intestinal parasites. Data were analyzed by SPSS version 21. Logistic regression analyses were performed and p-value <0.05 was considered as statistically significant.

**Results:**

The overall prevalence of anemia among dyspeptic patients was 24.3% (95%CI: 19.9–28.7). Among *H*.*pylori* infected participants 29.2% were anemic, of which 69.2% had mild anemia and 63.5% had normocytic normochromic anemia. Rural residence (AOR: 1.9, 95%CI: 1.1–3.3), *H*. *pylori* infection (AOR: 1.77, 95%CI: 1.05–2.98) and intestinal parasitic infection (AOR: 2.14, 95%CI: 1.14–4.03) were significantly associated with anemia.

**Conclusion:**

The prevalence of anemia in this study indicated that it is a moderate public health problem. Rural residence, *H*. *pylori* and intestinal parasitic infection were significantly associated with anemia. The findings of this study should be taken into account for the prevention and control of anemia among dyspeptic adults.

## Introduction

Anemia is a global public health problem affecting both developed and developing countries [1]. A study reported a 32.9% global burden of anemia in all ages combined, Sub-Saharan Africa shared the highest-burden [2]. It has serious implications for health, as well as social and economic development [3]. It increases susceptibility to infections, impairs the physical capacity and work performance of the adults [4, 5]. It is a significant public health problem in Ethiopia, according to a national report of 2016, 24% of women and 15% of men were anemic [6].

Identifying possible contributing factors of anemia at different setups is important to combat its burden and for proper management of anemic patients. Several studies identified different factors associated with anemia among adults [7–9]. Recently *H*. *pylori* infection has been implicated in some of the hematological manifestations like anemia, iron and vitamin B12 deficiency [10–12].

*H*. *pylori* infection is associated with anemia by impairing iron absorption as a result of chronic gastritis which causes gastric hypochlorhydria, leading to impair reduction of the dietary iron from the ferric to ferrous form [13, 14]. Because most dietary iron is in the ferric form, and an acidic intragastric pH and ascorbic acid are needed to reduce it to the ferrous form for absorption [15]. Hence, *H*. *pylori* is a major cause of chronic superficial gastritis leading to atrophy of gastric glands, resulting in reducing gastric acid secretion [16].

*H*. *pylori* uptakes iron for their growth by competing with their host [4], and increased hepcidin production secondary to *H*. *pylori* infection decreases the release of iron from macrophages and entrecote [17]. Because hepcidin, act as an acute-phase reactant in response to the inflammation produced in the gastric mucosa, resulting in anemia of inflammation or chronic disease [14]. Other possible mechanism includes iron loss via hemorrhagic gastritis and active bleeding peptic ulcers [18].

*H*. *pylori* infection is a prevalent microbial infection around the world, 48.6% of the adult population were affected globally [19]. It has been found more frequently in dyspeptic patients, studies reported 71%, 72.2% and 83.3% in Somali [20], Southern [21] and Northern Ethiopia [22], respectively.

In Ethiopia data regarding the association of *H*.*pylori* infection with anemia among adults is scarce, despite a high prevalence of this infection [20–23]. Therefore disclosing the association of *H*. *pylori* infection with anemia among adult dyspeptic patients will help to develop evidence-based decision and intervention strategies. The availability of updated data on the magnitude, type, and severity of anemia has a major role in the management of anemic patients. Therefore, this study aimed to determine the prevalence, type, and severity of anemia and its association with *H*. *pylori* infection among adult dyspeptic patients in Southwest Ethiopia.

## Materials and methods

### Study area

This study was conducted at Wachemo University Nigist Eleni Mohammed Memorial Referral Hospital (WUNEMMRH). The hospital is located in Hosanna town, Hadiya Zone, Southern Nations Nationalities, and People’s Regional state, Southwest Ethiopia. The town is 232 km far from capital-city, Addis Ababa and lies in on average at 2,177 meters above sea level. The hospital gives health services for peoples living in the Hadiya zone and surrounding districts. Its catchment area population estimated around 3.2 million.

### Study design and period

The facility-based cross-sectional study design was conducted among adult dyspeptic patients from January 2019 to April 2019.

### Sample size determination and sampling technique

The sample size was determined by using a single population proportion formula considering a 95% confidence interval (CI), a 5% margin of error and a 30.9% prevalence of anemia [24]. After adding (10%) non-response rate we got the final sample size of 362. All consecutively identified adult dyspeptic patients (age ≥18years) who have dyspeptic complaints were included in the study until attaining sample size. Patients who took treatment for *H*. *pylori* infection within last three month [25], who had previous stomach or small bowel surgery, donate blood within last three month and on treatment for anemia before data collection, pregnant women and severally ill patients thus unable to respond to the questionnaire were excluded.

### Data collection and laboratory procedure

Data on socio-demographic characteristics, clinical and other related factors were collected using a structured questionnaire by trained nurses. For laboratory data, 4ml of the venous blood sample was collected in ethylenediaminetetraacetic acid (EDTA) tubes by laboratory technologists from each dyspeptic patients for hematological parameter analysis and blood film preparation. Hematological parameters were determined using automated blood analyzer Mindray BC-3000 plus (Shenzhen Mindray Bio-Medical Electronics, China).

Anemia in adult dyspeptic patients was defined according to the World health organization (WHO) cutoff value as an HGB concentration <12 g/dl in women and < 13 g/dl in men. It is categorized as mild (HGB,11–11.9 g/dl in women and 11–12.9 g/dl in men), moderate (HGB, 8–10.9 g/dl in both men and women), and severe anemia (HGB, < 8 g/dl in both men and women) [26]. It can be classified as microcytic (MCV, <80 fl), normocytic (MCV, 80 fl-100 fl) and macrocytic (MCV, >100 fl) [27, 28]. Thin blood films were prepared, air-dried, labeled and then stained by wrights stain to evaluate RBC morphology of anemic study participants.

Also, after explaining how to collect representative stool specimen clean cupped plastic container was given to the participants. Approximately five gram of stool specimen was collected from each dyspeptic patients and checked for the presence of *H*. *pylori* antigen by wondfo one step *H*. *pylori* feces test (Guangzhou Wondfo Biotech, China) and intestinal parasites were detected by saline wet mount techniques. Anthropometric measurements (height and weight) were measured from all study participants and body mass index (BMI) was computed as weight in kilogram divided by the square of height in meter and categorized in a four groups; BMI<18.5 kg/m^2^ as underweight, BMI = 18.5–24.9 kg/m^2^ as normal weight, BMI = 25–29.9 kg/m^2^ as overweight, and BMI ≥ 30 kg/m^2^ as obese [29].

To ensure the quality of data, training was given to data collectors, completeness of each questionnaire was checked regularly, reagents and test kits were checked for their expiry date. All laboratory tests were done by following the standard operating procedures (SOPs) and manufacturer instructions.

### Data analysis and interpretation

Data were entered and analyzed by using SPSS version 21 (SPSS, Chicago, IL, USA). Frequency tables and descriptive summaries were used to describe the study variables. Association between anemia and *H*. *pylori* infection was assessed by a logistic regression model. Bivariate analysis was performed for each independent variable to select candidate’s variables for multivariate analysis. Variables in bivariate analysis with P-value <0.25 were taken as candidates for multivariate analysis. Multiple logistic regression analysis was used to identify associated risk factors for the prevalence of anemia in dyspeptic patients. P-value was set at <0.05 for statistical significance.

### Ethical considerations

Ethical clearance was obtained from Jimma University Ethical Review Board. A letter of cooperation was written to WUNEMMRH and permission was obtained from the hospital administration. Written informed consent was obtained from each study participant after explaining the purpose and procedures of the study.

The study participant results were kept confidential and they were assured that only aggregate data will be reported. All necessary results of the participant were communicated with the physician for proper management.

## Results

### Socio-demographic, clinical and related characteristics of the study participants

A total of 362 adult dyspeptic patients, 58% females and 42% males were included in this study age ranging from 18 to 49 years, with a mean (±SD) age of 31.1(±7.5) years. The majorities of them were rural area residents 57.7% and married 60.2%. *H*. *pylori* infection and intestinal parasites were detected in 49.2% and 16.3% of the study participants, respectively. Among the study participants, 2.5% had chronic disease and 3% had a history of bleedings. The majority of 61.6% of participants had a habit of consumption of fruit and vegetable during the time of the data collection (Table 1).

**Table 1:** Socio demographic, clinical and other related characteristics of study participants.

Variables	Categories	n (%)
**Age in years**	18–24	85(23.5)
	25–29	91(25.1)
	30–34	59(16.3)
	35–39	56(15.5)
	40–44	54(14.9)
	45–49	17(4.7)
**Gender**	Female	210(58)
	Male	152(42)
**Residence**	Urban	153(42.3)
	Rural	209(57.7)
**Marital status**	Single	136(37.6)
	Married	218(60.2)
	Divorced	4(1.1)
	Widowed	4(1.1)
**Educational status**	Illiterate	99(27.3)
	Primary	50(13.8)
	Secondary	115(31.8)
	Higher	98(27.1)
Monthly income in ETB*	<776	69(19.1)
	776–1552	97(26.8)
	>1552	196(54.1)
**Occupational status**	Farmer	94(26)
	Daily laborer	55(15.2)
	Employee	80 (22.1)
	Students	69(19.1)
	Merchants	57(15.7)
	Self employee	7(1.9)
**Intestinal parasite**	Positive	59(16.3)
	Negative	303(83.7)
**Chronic disease**	Yes	9(2.5)
	No	353(97.5)
**History of bleedings**	Yes	11(3)
	No	351(97)
**BMI in kg/m**^**2**^	Underweight	11(3)
	Normal weight	303(83.7)
	Overweight	48(13.3)
**Fruit/vegetable consumption**	Yes	223(61.6)
	No	139(38.4)
**Red meat consumption**	Yes	123(34)
	No	239(66)

*ETB: Ethiopian Birr.

### Prevalence, severity, and types of anemia among adult dyspeptic patients

The mean (±SD) HGB concentration of the study participants was 13.5(1.93) g/dl in females and 14.1(1.55) g/dl in males. The overall prevalence of anemia among dyspeptic patients was 24.3% with 95%CI (19.9–28.7). Of which 71.6% had mild and 28.4% moderate anemia but there was no severe anemia identified. Out of anemic participants, 27.3%, 5%, and 2.3% had microcytic, normocytic and macrocytic types of anemia, respectively.

### Prevalence, severity, types, and association of anemia with *H*. *pylori* infection

The prevalence of anemia among *H*. *pylori* infected participants was 29.2%, of which 69.2%, 30.8% had mild and moderate anemia, respectively. Among *H*.*pylori* infected study participants, 34.6%, 63.5%, and 1.9% had microcytic, normocytic and macrocytic anemia, respectively. After adjusting for other variables, *H*. *pylori* infection showed a statistically significant association with anemia (Table 2).

**Table 2:** Factors associated with anemia among study participants at Wachemo University Nigist Eleni Mohammed Memorial Referral Hospital, 2019 (n = 362).

Variables	Categories	Anemia		COR(95%CI)	AOR(95%CI)
		Yes, n (%)	No, n (%)		
**Residence**	Urban	26(17)	127(83)	1	1
	Rural	62(29.7)	147(70.3)	2.06(1.23–3.45)	1.9(1.1–3.3)*
**Educational status**	Illiterate	31(31.3)	68(68.7)	1.89(0.98–3.65)	1.66(0.82–3.3)
	Primary	15(30)	35(70)	1.78(0.81–3.9)	1.54(0.67–3.5)
	Secondary	23(20)	92(80)	1.03(0.52–2.04)	1.12(0.55–2.27)
	Higher	19(19.4)	79(80.6)	1	1
***H*. *Pylori* antigen test**	Positive	52(29.2)	126(70.8)	1.69(1.04–2.76)	1.77(1.05–2.98)*
	Negative	36(19.6)	148(80.4)	1	1
**Intestinal parasite**	Positive	21(35.6)	38(64.4)	1.94(1.07–3.54)	2.14(1.14–4.03)*
	Negative	67(22.1)	236(77.9)	1	1
**BMI in kg/m**^**2**^	Underweight	5(45.5)	6(54.5)	3.16(0.8–12.5)	2.88(0.68–12.1)
	Normal	73(24.1)	230(75.9)	1.2(0.57–2.54)	1.29(0.59–2.8)
	Overweight	10(20.8)	38(79.2)	1	1
**Chronic disease**	Yes	4(44.4)	5(55.6)	2.56(0.67–9.7)	1.87(0.46–7.59)
	No	84(23.8)	269(76.2)	1	1
**History of bleeding**	Yes	5(45.5)	6(54.5)	2.69(0.8–9.04)	3(0.84–10.76)
	No	83(23.6)	268(76.4)	1	1

AOR: adjusted odds ratio; COR: crude odds ratio; CI: confidence interval; 1: reference;

*: statistically significant association (p -value <0.05).

### Factors associated with anemia

After adjusting for other variables: rural residence (AOR: 1.9, 95%CI: 1.1–3.3), *H*. *pylori* infection (AOR: 1.77, 95%CI: 1.05–2.98) and intestinal parasitic infection (AOR: 2.14, 95%CI: 1.14–4.03) were significantly associated factors with anemia among study participants (Table 2).

## Discussion

The current study attempted to asses anemia prevalence and its association with *H*. *pylori* infection among adult dyspeptic patients. The overall prevalence of anemia among adult dyspeptic patients was 24.3%. According to the WHO classification of the public health importance of anemia [26], anemia prevalence among adult dyspeptic patients in this study indicated a moderate public health problem.

The overall prevalence of anemia among adult dyspeptic patients obtained in this study was consistent with a study done in Butajira, Ethiopia (26.9%) [24], Cuba (24.6%) [30], Karachi (25.2%) [31] and Southern Brazil 20.6% [32].

The prevalence of anemia obtained among *H*.*pylori* infected adult dyspeptic patients in this study was 29.2%, which was consistent with the study finding in Butajira, Ethiopia 30.9% [24], while higher magnitudes of anemia among *H*.*pylori* infected were reported from Kutahya, Turkey (91.8%) [33] and Central Plateau (55.2%) [34].

According to WHO classification for the degree of anemia based on HGB concentration [26], in this study, mild anemia was common (69.2%) followed by moderate anemia (30.8%) among *H*.*pylori* infected dyspeptic patients. Similar findings were reported in studies done in China [35] and Cuba [30].

Considering the morphological classification of anemia, normocytic-normochromic anemia (63.5%) was the predominant type of anemia among *H*.*pylori* infected dyspeptic patients followed by microcytic hypochromic anemia (34.6%) in this study. This might be due to the reason that blood loss secondary to chronic erosive gastritis, decreased iron absorption secondary to chronic gastritis and hypochlorhydria, and also rises in hepcidin level after *H*. *pylori* infection which might contribute in anemia [11, 14].

The association between anemia and *H*. *pylori* infection among adult dyspeptic patients has been explored by the number of previous studies [24, 30, 32–35]. The current study revealed that there is a statistically significant association between anemia and *H*. *pylori* infection in adult dyspeptic patients. *H*. *pylori*-infected individuals were 1.77 times more likely to be anemic compared to their non-infected counterparts. These findings were in agreement with previous studies conducted in China [35, 36] and the USA [37]. Also, different studies had reported similar findings [10, 38–40].

The possible mechanism that might explain the association between anemia and *H*. *pylori* infection among adult dyspeptic patients may include; consumption of iron by the organism itself [41], gastrointestinal blood loss due to *H*. *pylori*-induced gastrointestinal lesions [14], and gastritis increased levels of neutrophil-derived lactoferrin, and since *H*. *pylori* has a lactoferrin-binding protein receptor, the infection may result in increased iron losses related to bacterial turnover. Since these bacteria have a high turnover rate, a large amount of iron may be lost in stools in the form of dead bacteria [11, 42]. Also, *H*. *pylori* chronic gastritis can change the physiology of the stomach, inducing reductions in gastric acid secretion, while acidic intragastric PH was essential for the absorption of dietary iron; thereby inhibiting dietary iron absorption [12].

Gastrointestinal blood loss is one of the most important underlying causes of anemia in adults [43–46]. In this study, anemia was significantly associated with intestinal parasitic infection. Study participants who had intestinal parasitic infection were 2.14 times more likely to be anemic compared to their non-infected counterparts. A similar observation was reported from Uganda [8] and India [9]. This might be due to gastrointestinal blood loss which may contribute to anemia. The worm in the intestine may cause blood loss as a result of the attachment to the intestinal mucosa and chronic infections may lead to iron deficiency and anemia resulting from the excessive loss of iron [44, 45].

Socio-demographic factors can play a role in determining anemia [7]. In the current study, anemia was significantly associated with rural residence. This finding is in agreement with the study done in India [7, 43]. This might be likely related to a lack of information about adequate nutrition and inaccessibility of health care centers. Thus, they lack information on the causes of anemia and possible prevention strategies to risk factors of anemia.

Despite the fact that the necessary endeavors were made to minimize or avoid the possible limitations of this study, our study results should be interperated under the concederation of following limitations; micronutrient (serum iron status, foliate and vitamin-B12) levels were not assessed and intestinal parasites were not detected by concentration techniques due to logistic constraints. Inflammatory markers such as C-reactive protein or acid glycoprotein α-1, leukocytes, neutrophils, and lymphocytes were not measured due logistic constraints. In addition since, the study was conducted in symptomatic adult patients with dyspepsia, which cannot be generalized to the asymptomatic adults with *H*. *pylori* infection. The cross-sectional nature of the study design prohibited to establish causal links between anemia and factors which are significantly associated with anemia.

## Conclusion

The prevalence of anemia in this study indicated that it is a moderate public health problem. A higher prevalence of anemia was observed in study participants having intestinal parasitic infection, *H*. *pylori* infection and resides in a rural area. Rural residence, *H*. *pylori* and intestinal parasitic infection were significantly associated with anemia. The findings of this study should be taken into account for developing intervention-based strategies on identified factors mainly on; prevention and control of *H*. *pylori* and intestinal parasitic infection. Routine screening and treatments of *H*. *pylori* and intestinal parasitic infection among dyspeptic adults, and performing large community-based studies are recommended.

## Supporting information

S1 File (DOCX)
