# Supplementary material for: Anemia and its association with Helicobacter pylori infection among adult dyspeptic patients attending Wachemo University Nigist Eleni Mohammad Memorial Referral Hospital, Southwest Ethiopia: A cross-sectional study
Source: PLoS One. 2021 Jan 14;16(1):e0245168. doi: 10.1371/journal.pone.0245168 (PMC7808578; doi:10.1371/journal.pone.0245168)
Supplement: S1 File — (DOCX) [file pone.0245168.s001.docx]

### English version questionnaire

Card no: ___________________________

Code: _______________________________

Address: _____________________________

**General instruction**

1. For all questions that have a pre- coded response,

- Circle the responses that best match with your response

For open ended questions write your responses in blank space

| SN | Variables | Response | Skip to |
| --- | --- | --- | --- |
| **Part I: Socio-Demographic characteristics** | | |  |
| 1.1 | Age in full year | _______________ |  |
| 1.2 | Sex | 1. Female 2. Male |  |
| 1.3 | Residence | 1. Urban 2. Rural |  |
| 1.4 | Marital status | 1. Single 2. Married 3. Divorced 4. Widowed |  |
| 1.5 | Educational status | ______________ |  |
| 1.6 | Monthly income in Ethiopian birr | ______________ |  |
| 1.7 | Occupational status | 1. Farmer 2. Daily labourer 3. Governmental employee 4. Students 5. Merchants 6. Self-employee 7. Other specify_______ |  |
| **Part II: clinical character** | | |  |
| 2.1 | Do you have known chronic illness like? |  | If No skip to 2.2 |
|  | Diabetes mellitus | 1. No 2. Yes |  |
|  | chronic kidney disease | 1. No 2. Yes |  |
|  | TB | 1. No 2. Yes |  |
|  | Hepatitis | 1. No 2. Yes |  |
|  | If other Specify | ___________ |  |
| 2.2 | Do you have history of bleedings? | 1. No 2. Yes | If No skip to 3.1 |
| 2.3 | If yes to Q.2.4. What is reason for bleeding? | 1. accidents 2. haemorrhoids 3. heavy menstruation 4. other specify ______ |  |
| **Part III : Dietary habits** | | | |
| 3.1 | Do you consume fruit and vegetable? | 1. No 2. Yes | If No skip to 3.3 |
| 3.2 | If yes for Q.3.1. How many days per week in average? | 1. 1 2. 2 3. 3 4. other specify ______ |  |
| 3.3 | Do you consume red meat? | 1. No 2. Yes | If No skip to 4.1 |
| 3.4 | If yes for Q.3.3**.** How many days per week in average? | 1. 1 2. 2 3. 3 4. other specify ________ |  |
| **Anthropometric measurements** | | |  |
| 4.1 | Weight (to be measured by data collectors) | ____________________kg |  |
| 4.2 | Height (to be measured by data collectors) | ____________________meter |  |
